# Comparison of surgical versus diet‐induced weight loss on appetite regulation and metabolic health outcomes

**DOI:** 10.14814/phy2.14048

**Published:** 2019-03-29

**Authors:** Tanya M. Halliday, Sarit Polsky, Jonathan A. Schoen, Kristina T. Legget, Jason R. Tregellas, Kayla M. Williamson, Marc‐Andre Cornier

**Affiliations:** ^1^ Division of Endocrinology, Metabolism and Diabetes University of Colorado School of Medicine Anschutz Medical Campus Aurora Colorado; ^2^ Department of Health, Kinesiology, and Recreation University of Utah Salt Lake City Utah; ^3^ Barbara Davis Center for Diabetes University of Colorado School of Medicine Anschutz Medical Campus Aurora Colorado; ^4^ Division of GI, Tumor and Endocrine Surgery University of Colorado School of Medicine Anschutz Medical Campus Aurora Colorado; ^5^ Division of Psychiatry University of Colorado School of Medicine Anschutz Medical Campus Aurora Colorado; ^6^ Department of Biostatistics and Informatics Colorado School of Public Health University of Colorado Anschutz Medical Campus Aurora Colorado; ^7^ Anschutz Health and Wellness Center University of Colorado School of Medicine Anschutz Medical Campus Aurora Colorado

**Keywords:** Appetite, diet‐induced weight loss, energy intake, gut peptides, roux‐en‐y gastric bypass

## Abstract

Bariatric surgery is associated with significant and sustained weight loss and improved metabolic outcomes. It is unclear if weight loss alone is the main mechanism of improved metabolic health. The purpose of this trial was to compare indices of appetite regulation, insulin sensitivity and energy intake (EI) between participants achieving 10 kg of weight loss via Roux‐en‐Y Gastric Bypass (RYGB) or dietary restriction (DIET); intake of a very low calorie liquid diet (800 kcal/d; 40% protein, 40% fat, 20% carbohydrate that matched the post‐RYGB dietary protocol). Adults qualifying for bariatric surgery were studied before and after 10 kg of weight loss (RYGB [*n* = 6]) or DIET [*n* = 17]). Appetite (hunger, satiety, and prospective food consumption [PFC]), appetite–related hormones, and metabolites (ghrelin, PYY, GLP‐1, insulin, glucose, free fatty acids [FFA], and triglycerides [TG]) were measured in the fasting state and every 30 min for 180 min following breakfast. Participants were provided lunch to evaluate acute *ad libitum* EI, which was similarly reduced in both groups from pre to post weight loss. Fasting ghrelin was reduced to a greater extent following RYGB compared to DIET (*P* = 0.04). Area under the curve (AUC) for ghrelin (*P* = 0.01), hunger (*P* < 0.01) and PFC (*P* < 0.01) increased after DIET compared to RYGB, following 10 kg weight loss. Satiety AUC increased after RYGB and decreased after DIET (*P* < 0.01). Glucose and insulin (fasting and AUC) decreased in both groups. FFA increased in both groups, with a greater increase in AUC seen after RYGB versus DIET (*P* = 0.02). In summary, appetite–related indices were altered in a manner that, if maintained, may promote a sustained reduction in energy intake with RYGB compared to DIET. Future work with a larger sample size and longer follow‐up will be important to confirm and extend these findings.

## Introduction

Obesity continues to be a significant public health issue affecting over one‐third of adults in the United States (Ogden et al. 2015). Due to increased risks of adverse health outcomes and associated medical costs (Finkelstein et al. 2009), weight loss is indicated for overweight/obese adults. While challenging, weight loss can be accomplished by a variety of lifestyle interventions (Wadden 1993; Hassan et al. 2016). However, weight loss and decreased energy intake result in physiologic and behavioral adaptations to restore homeostasis by encouraging increased energy intake to oppose weight loss and promote weight regain (Maclean et al. 2011; Ochner et al. 2013; Melby et al. 2017). Thus, the more difficult aspect of weight management is for individuals to maintain the weight–reduced state long‐term (Maclean et al. 2011; MacLean et al. 2015).

Bariatric surgery procedures, specifically Roux‐en‐Y Gastric Bypass (Park and Torquati 2011) (RYGB), are associated with greater success at long‐term weight loss maintenance and sustained improvements in obesity–related comorbidities as compared to non‐surgical weight loss strategies (Falken et al. 2011; Gloy et al. 2013; Sjostrom 2013; Maciejewski et al. 2016). While the mechanisms are not fully elucidated, changes in gastrointestinal anatomy such as decreased gastric capacity, altered nutrient transit, and changes in gastrointestinal cell exposure to ingested food/nutrients lead to physiological alterations (e.g., altered metabolite and appetite–related hormone response profiles) that are established factors in weight and health–related improvements following RYGB (le Roux et al. 2006; Park and Torquati 2011; Quercia et al. 2014). Despite these physiological alterations, long‐term weight loss maintenance requires substantial and sustained changes in eating related behaviors. RYGB has also been associated with changes in food preferences away from high‐calorie and highly hedonic choices that would promote decreased energy intake (Behary and Miras 2015; Manning et al. 2015). Thus it has been suggested that RYGB leads to a “resetting” of the appetite–related hormonal response, for example lower ghrelin and greater peptide YY (PYY) and glucagon–like peptide‐1 (GLP‐1) levels in response to a meal postsurgery as compared to presurgery (Cummings et al. 2002; Morinigo et al. 2006, 2008; le Roux et al. 2006, 2007; Rodieux et al. 2008; Harvey et al. 2010; Schmidt et al. 2016; Steinert et al. 2017) which contributes to both beneficial changes in homeostatic appetite control, and in hedonic food responses (Saper et al. 2002; Gibson et al. 2010; Ochner et al. 2011; Evans et al. 2012; Scholtz et al. 2014). Ultimately this would increase the likelihood of long‐term adherence to decreased energy intake and contribute to successful weight loss maintenance and improvements in metabolic health.

This area of investigation is still emerging, and questions remain regarding if and how changes in appetite–related peptides with RYGB are linked to changes in subjective appetite, food cravings, and food intake (Diniz Mde et al. 2010). Furthermore, research in this area is limited by study designs that are not well‐suited to answer these questions, nor to compare RYGB to diet‐induced weight loss. For instance, prior studies have not been prospective, have utilized nonobese adults as the comparison group for RYGB, and/or have not studied RYGB and diet‐induced weight loss participants at matched levels of weight loss (Khoo et al. 2014; Goldstone et al. 2016; Schmidt et al. 2016). To address these gaps in the literature, the purpose of the current trial was to compare the influence of weight loss method (RYGB vs. very low calorie diet (VLCD)–induced weight loss) on changes in appetite regulation and biomarkers of metabolic health. Specifically, we aimed to evaluate how matched, 10 kg of weight loss via these two methods affects appetite‐related peptides, appetite ratings, food cravings, energy intake, and measures of glucose homeostasis. We hypothesized that RYGB will result in changes to appetitive indices in a manner supportive of reduced energy intake (e.g., – decreased ghrelin and increased PYY and GLP‐1) as well as superior alterations to metabolite profiles (e.g., – decreased glucose, insulin, and triglycerides) as compared to VLCD–induced weight loss.

## Materials and Methods

### Participants

Patients undergoing evaluation for RYGB at the University of Colorado's Surgical Weight Loss Center were recruited for enrollment in the current trial. Participants were recruited for inclusion in the diet‐induced weight loss control group (DIET) via advertisements for enrollment in a diet‐induced weight loss intervention. To be eligible for inclusion, participants were required to: be between the ages of 21–65 years ; meet National Institutes of Health criteria for gastric bypass surgery (BMI > 40 kg/m^2^ or BMI ≥ 35 kg/m^2^ with high‐risk comorbid conditions) (Consensus Panel, 1992); be weight‐stable (±5%) over previous 6 months; be free of significant uncontrolled medical illness (e.g., uncontrolled diabetes, untreated severe dyslipidemia, uncontrolled hypertension, uncontrolled hyper or hypothyroidism, gastrointestinal disorders influencing food intake, or cancer) and/or major psychiatric disorder; and not be a current smoker or alcohol/substance abuser (by self‐report). In addition, participants were excluded if they: were taking medications known to influence metabolism, body weight, energy expenditure, or appetite; were currently pregnant, lactating, or < 6 months postpartum; or had a history of depression (or a score > 21 on the CES‐D [Radloff 1977]) or eating disorders (or score > 20 on the EATS‐26 [Garner et al. 1982]).

Patients who elected to undergo the surgical procedure and complete study–associated testing visits were included in the RYGB group (*n* = 6). Individuals who responded to study advertisements for the diet‐induced weight loss intervention and met inclusion criteria were included in the DIET group (*n* = 17). The Colorado Multiple Institutional Review Board approved the study protocol and all participants provided written informed consent prior to participation in study‐associated procedures. This study was conducted in accordance with the principles expressed in the Declaration of Helsinki. Study data were collected and managed using REDCap electronic data capture tools hosted at the University of Colorado Anschutz Medical Campus (Harris et al. 2009).

### Study design and measurements

Following screening and written informed consent, subjects completed baseline evaluations including: height (without shoes to the nearest cm using a stadiometer), weight (in light clothing to the nearest 0.1 kg using a digital scale); assessment of body composition via dual energy X‐ray absorptiometry (DEXA; Delphi‐W version 11.2; Hologic Inc., Bedford, MA); completion of the original version of the Three Factor Eating Inventory Questionnaire (TFEI‐Q) (Stunkard and Messick 1985); and completion of a Food Frequency Questionnaire (FFQ) to evaluate habitual food intake over the prior 12‐months (via the National Cancer Institute's Diet History Questionnaire II [National Institutes of Health EaGRP, 2010]). Each participant then completed a 3‐day run‐in dietary control period and underwent the baseline study day visit (described below) to evaluate behavioral and hormonal measures of appetite regulation as well as insulin sensitivity, followed by either RYGB for the surgical group or a VLCD for the DIET group. Participants were seen in our outpatient Clinical and Translational Research Center (CTRC) every other week to ensure safety and track body weight. Participants then repeated body mass measures and the study day visit when ~10 kg of weight loss was achieved.

#### Roux‐en‐Y gastric bypass protocol

Following baseline measurements, participants in the RYGB group underwent laparoscopic RYGB as per standard procedures in the University of Colorado's Surgical Weight Loss Center. Briefly, an endoGIA (Medtronic, Minneapolis, MN) linear stapler was used to create a 20 cc lesser curvature gastric pouch. The proximal jejunum was measured 25 cm from the ligament of treitz and divided with the endoGIA linear stapler and the Roux limb was measured at either 100 cm (for BMI < 50) or 150 cm (for BMI > 50) and placed in an antecolic antegastric position. The gastrojejunostomy and jejunojejunostomy were created with the linear stapler as well. All mesenteric defects were closed with permanent sutures. There were no acute complications and all patients were discharged between postoperative days 2 and 3. One patient developed a mild infection at an incision site on postoperative day 16. Post‐operative nutritional needs were met as per usual care in the University of Colorado's Surgical Weight Loss Center. Briefly, patients progressed from a post‐operative clear liquid diet to a standard post‐RYGB low‐calorie (800 kcal/d) liquid diet with a macronutrient composition of 40% protein, 40% fat, and 20% carbohydrates. All participants in the RYGB group were enrolled in the trial within 2 months prior to surgery, and underwent surgery within 2 weeks of completing baseline measures. Time to weight loss was measured from postoperative day 1. Importantly, body mass was stable between enrollment, baseline testing, and surgery (<±3%).

#### Diet‐induced weight loss protocol

Following baseline measurements, participants in the DIET group began a VLCD designed to be consistent with the nutritional needs of the RYGB group. Diets were provided by the CTRC Metabolic Kitchen and consisted of an 800 kcal/day liquid diet (flavored shakes) with a macronutrient composition of 40% protein, 40% fat, and 20% carbohydrate. All participants in the DIET group underwent baseline testing within 6 weeks of enrollment in the trial and initiated the VLCD the day after baseline testing.

#### Run‐in diet

To ensure weight maintenance and both energy and macronutrient balance, participants consumed a controlled eucaloric diet for 3 days prior to the baseline study day visit. The caloric value of the diet was individualized for each participant and determined using lean body mass (as determined by DEXA) in the following equation: Resting Metabolic Rate (RMR) = (fat free mass*23.9) + 372, and multiplying by a correction factor of 1.4. This method has been used successfully by our group to maintain energy balance in a number of prior studies (Cornier et al. 2004, 2006, 2007, 2009; Adochio et al. 2009; Tregellas et al. 2011). The run‐in diet had a macronutrient composition of 40% protein, 40% fat, and 20% carbohydrate in order to mimic post‐RYGB nutritional recommendations. All food was prepared and provided by the CTRC metabolic kitchen. Subjects presented to the CTRC every morning, ate breakfast, and picked up the remainder of their daily meals. To increase adherence, specific foods provided consisted of commonly consumed items that were tailored to each subject based upon a food preference survey administered by the CTRC metabolic kitchen. Subjects were asked to maintain their usual pattern of physical activity and were regularly questioned regarding activity and dietary compliance. Since participants were in active weight loss prior to the follow‐up study visit at 10 kg of weight loss, the run‐in diet period was not repeated.

#### Study day visit

At baseline and at 10 kg of weight loss, participants presented to the CTRC after an overnight fast of at least 10 h for the following study day visit procedures. Participants were weighed, had an intravenous (IV) catheter placed for serial blood sampling, completed fasting appetite, food craving, and visual food stimuli evaluations, and had a fasting blood draw to determine appetite–related hormone and metabolite levels. Participants then consumed a standard liquid breakfast meal (chocolate or vanilla shake prepared by the CTRC Metabolic Kitchen) over 20 min that contained 25% of estimated total daily energy requirements at each time point with a macronutrient composition identical to the run‐in diet. This insured provision of the same “relative” caloric load (25%) at each time point. Repeat blood sampling and appetite evaluations were conducted at 30, 60, 90, 120, 150, and 180 min following the test meal. Food craving questionnaire and visual stimuli evaluations were also repeated in the fed state. Following the 180‐min sampling period, the IV catheter was removed and participants consumed an *ad libitum* lunch meal (described below).

##### Laboratory analysis

Blood samples were collected in EDTA–containing tubes, centrifuged, placed in aliquot tubes and stored at −70 to −80°C until post trial batch (with samples from each participant run in the same assay) analysis of metabolites (insulin, glucose, free fatty acids [FFA], and triglycerides [TG]) and appetite–related hormones (total ghrelin, total PYY, total GLP‐1, and leptin). With the exception of leptin, which was only measured in the fasting state, the area under the curve (AUC) for all laboratory measures was calculated using the trapezoid method (Allison et al. 1995). For GLP‐1, 30 *μ*L of dipeptidyl peptidase IV inhibitor was added to the 4 mL EDTA tube prior to collection. GLP‐1 assays were performed with Alpco Diagnostics ELISA (43‐GPTHU‐E01). Insulin concentrations were measured using competitive radioimmunoassay (Millipore). Radioimmunoassay was used to analyze serum leptin (Millipore), serum PYY concentrations (Millipore Cat. #PYYT‐66HK) and total serum ghrelin concentrations (Millipore Cat. #GHRT‐89HK). All radioimmunoassays were performed with a Perkin Elmer Wallac Gamma counter using Maciel RIA‐AID data reduction software. Assays for glucose, TG and FFA were performed on the Olympus AU400e Chemistry Analyzer (Beckman). Reagents were purchased from Beckman Coulter for glucose and TG and from WACO for FFA.

##### Appetite evaluations

Participants completed subjective appetite ratings measured using the visual analogue scale (VAS) on a handheld computerized device for hunger, satiety, and prospective food consumption (PFC). Hunger was rated on a 100‐mm line preceded by the question, “How hungry do you feel right now?” and anchored on the left by “not at all hungry” and by “extremely hungry” on the right. Satiety was rated by the question, “How full do you feel right now?” with the anchors “not at all” and “extremely” (Cornier et al. 2004). PFC was rated by the question, “How much food do you think you could eat right now?” with the anchors “nothing at all” and “a large amount”. The AUC for all appetite ratings was calculated using the trapezoid method (Allison et al. 1995).

##### Food cravings questionnaire

Participants also completed the Food Craving Inventory (FCI) (White et al. 2002) in both fasted and fed states on study days at both assessment time points. This tool assesses current food cravings on a 1–5 scale, with 1 associated with the text anchor “Strongly disagree” and 5 associated with the text anchor “Strongly agree”, using questions such as “I have an intense desire to eat one of my favorite foods”. Scores on each of the 15 items are summed, generating a scale that ranges from 15 to 75, with higher scores indicating a high food‐craving level.

##### Visual stimuli evaluations

In the fasted and fed state, participants were asked to rate food “appeal”, “pleasantness”, and “desire to eat” of previously validated low– and high–hedonic visual food stimuli (Burger et al. 2011). High‐hedonic items included pictures of appealing foods such as such as pizza, ice cream, and French fries. Low‐hedonic items included pictures of less appealing foods such as vegetables, bagels, and broth‐based soups. Food images were presented one at a time on a computer screen and participants assessed “appeal” “pleasantness” and “desire to eat” via a 100‐mm VAS depicted on the computer monitor underneath the image of each food. Appeal was rated by the question “How appealing is this food?” and anchored on the left by “not appealing at all” and by “extremely appealing” on the right. Pleasantness was rated by the question “How pleasant is this picture?” with the anchors, “not at all pleasant” and “extremely pleasant”. Desire to eat was rated by the question, “How much do you desire to eat this food?” with the anchors, “I have no desire to eat this food” and “I have a strong desire to eat this food.” A total of 96 food images (48 high‐hedonic and 48 low‐hedonic) were assessed by participants. Half of the images were presented in the fasted state, and half in the fed state, with an equal balance of high‐ and low‐hedonic images at each time point. VAS scale ratings were averaged across low‐ and high‐hedonic images for each question in both the fasted and fed state.

##### *Ad libitum* energy intake

Following the final blood draw and appetite ratings, participants were offered an *ad libitum* lunch in order to objectively evaluate energy intake. At baseline, a “buffet” style solid food meal totaling 1800 kcals (comprised of lasagna, salad with dressing, rolls, butter, pound cake, strawberries, and both diet and regular soda) was presented. Following 10 kg of weight loss, a 1500 kcal liquid shake with a macronutrient composition identical to their current dietary regimen (comprised of soy milk, water, whey protein powder, half and half, canola oil, French vanilla coffee creamer, polycose powder, and imitation vanilla flavor) was presented. In both conditions, participants were seated in a private room, were told they had 30 min to consume as much or as little of the meal as they wished, and were informed they could request more of any food. All food was prepared and provided by the CTRC metabolic kitchen. Energy intake was determined via the “weigh and measure” method by CTRC metabolic kitchen staff.

### Statistical analysis

Data were analyzed using R studio version 3.4.0 (2017‐04‐21) (R Core Team [2017]). A mixed effects analysis with a random slope was used to investigate whether the changes in measures between baseline and 10 kg weight loss differed between the DIET and RYGB groups. This was accomplished by including time, group, and time X group interaction term in the mixed effects model. A *t*‐test was used to assess the significance of the time X group interaction term. A separate model was fitted to each outcome. To assess sensitivity to the males in the DIET group, we repeated the above analysis removing the three males in the DIET group. We further investigated whether appetite ratings and food craving (FCI score) measures were correlated with *ad libitum* energy intake by adjusting for current appetite ratings or FCI scores in the above mixed model. The adjustment variable was a time varying covariate. We fitted a separate model for each measure given the moderate sample size in this study. All statistical tests were two‐tailed with significance set at *P* < 0.05 and data are reported as means and standard errors unless otherwise noted.

## Results

### Participant characteristics

A total of 23 participants were included in this trial with *n* = 6 in the RYGB group and *n* = 17 in the DIET group. Baseline participant characteristics and days required to achieve 10 kg of weight loss for each group are presented in Table 1 **.** The groups were well matched with respect to age, body composition, and habitual dietary intake. The RYGB group scored higher on the “dietary restraint” component of the TFEI‐Q than the DIET group (*P* = 0.02). Weight loss (~10 kg) was achieved more quickly in the RYGB group than the DIET group (*P* = 0.045).

**Table 1:** Baseline characteristics and time to achieve 10 kg of weight loss

	DIET Mean (SE)	RYGB Mean (SE)
Total *n* (male)	17 (3)	6 (0)
Age, years	41.3 (2.7)	37.6 (4.6)
BMI kg/m^2^	40.7 (0.9)	41.4 (1.4)
Percent body fat	45.4 (1.1)	48.2 (0.4)
Three factor eating inventory questionnaire (TFEI‐Q)		
TFEI‐Q – restraint	6.2 (0.9)	11.5 (1.6)*
TFEI‐Q – disinhibition	8.8 (1.0)	9.2 (1.5)
TFEI‐Q – hunger	6.7 (1.0)	7.2 (1.0)
Food frequency questionnaire		
FFQǂ – total energy/d, kcals	2185 (303)	1990 (277)
FFQǂ – total protein/d, %	15.3 (1.2)	15.4 (1.2)
FFQǂ – total fat/d, %	35.6 (1.6)	34.6 (4.3)
FFQǂ – total carbohydrate/d, %	49.5 (2.4)	51.4 (5.1)
FFQǂ – total alcohol/d, %	1.0 (0.4)	0.6 (0.6)
Weight loss		
Weight loss (kg)^a^	9.1 (0.4)	9.7 (0.7)
Weight loss (%)	8.2 (0.4)	9.2 (0.7)
Time to achieve 10 kg of weight loss (days)	46.4 (4.3)	30.0 (4.6)*

DIET, diet‐induced weight loss control group; RYGB, Roux‐en‐Y Gastric Bypass; BMI, body mass index; TFEI‐Q, three factor eating inventory questionnaire; FFQ, food frequency questionnaire; %: percent of total energy intake.

*P < 0.05; ^ǂ^FFQ data only available on *n* = 4 RYGB and *n* = 16 DIET participants.

a Weight loss did not equal 10 kg exactly due to scheduling. Weekly weights were used to estimate when ~10 kg of weight loss would be achieved and the follow‐up visits were scheduled in the CTRC accordingly.

### Appetite–related hormones and metabolites

**Figure 1:**
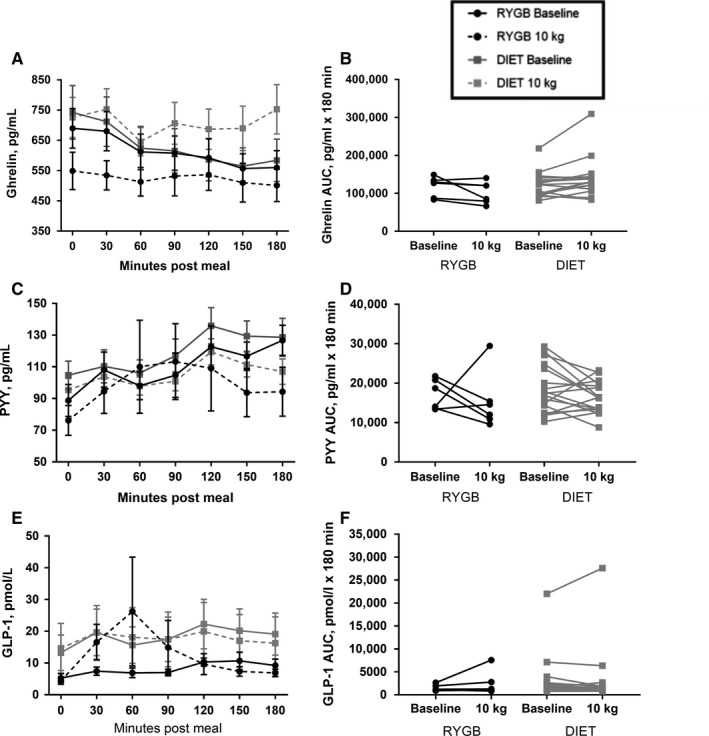
Appetite–related peptide response to breakfast test meal pre and post 10 kg of weight loss in participants undergoing RYGB or DIET. Curves for ghrelin (A), PYY (C), and GLP‐1 (E) are shown at 0 min and every 30 min for 180 min following the breakfast meal. Values are means ± SEM. Also shown are individual changes in AUC from pre to post 10 kg of weight loss for ghrelin (B), PYY (D), and GLP‐1 (F). RYGB, Roux‐en‐Y Gastric Bypass (*n* = 6); DIET, diet‐induced weight loss control group (*n* = 17); AUC, Area under the curve.

The group and individual AUC for appetite–related hormones (ghrelin, PYY, and GLP‐1) are presented in Figure 1. There was a significant interaction between group and time for both fasting ghrelin and ghrelin AUC (Table 2, *T* = −2.25, *P* = 0.04 and *T* = −2.53, *P* = 0.01, respectively), with the DIET group experiencing an increase in ghrelin AUC as compared to a decline in the RYGB group, and the RYGB group experiencing a greater decline in fasting ghrelin compared to DIET. No statistically significant differences were detected for fasting or AUC values for PYY or GLP‐1 between or within groups from pre to post 10 kg of weight loss (all *P* > 0.05). GLP‐1 findings were robust to removal of the outlier.

**Table 2:** Fasting and AUC hormone and appetite ratings, food craving inventory scores, and *ad libitum* energy intake at baseline and 10 kg of weight loss

	Baseline diet Mean (SE)	Baseline RYGB Mean (SE)	10 kg Diet Mean (SE)	10 kg RYGB: Mean (SE)	Treatment by time: Pr (>|*t*|)	Treatment by time: *T*‐value	Bonferroni Correction
Fasting values							
Ghrelin, pg/mL	742.2 (52.4)	689.7 (65.5)	725.7 (66.5)	548.8 (67.7)	0.04	−2.25	0.84
PYY, pg/mL	104.7 (8.8)	88.8 (10.1)	95.4 (7.3)	76.2 (9.5)	0.8	−0.25	1
GLP‐1, pmol/L	13.2 (5.6)	5.3 (1.4)	14.7 (7.8)	4.4 (1.1)	0.58	−0.56	1
Glucose, mg/dL	89.6 (2.0)	98 .0 (6.0)	85.8 (2.1)	89.8 (8.1)	0.25	−1.18	1
Insulin, *μ*IU/mL	28.5 (7.3)	27.0 (4.0)	15.5 (1.8)	14.7 (0.9)	0.96	0.06	1
FFA, *μ*Eq/L	645.2 (52.2)	739.7 (113.4)	850.8 (66.0)	1006.0 (61.1)	0.48	0.42	1
TG, mg/dL	107.7 (10.9)	105.7 (14.1)	89.4 (7.6)	94.2 (13.8)	0.7	0.39	1
Leptin, ng/mL	32.1 (4.3)	39.7 (7.1)	19.8 (1.9)	16.8 (1.6)	0.19	−1.35	1
Hunger, mm	78.6 (4.5)	69.4 (14.9)	64.1 (6.7)	50.5 (16.5)	0.65	−0.46	1
Satiety, mm	16.3 (6.2)	24.6 (17.2)	20.1 (4.8)	25.5 (9.6)	0.87	−0.17	1
PFC, mm	74.4 (3.2)	60.4 (13.8)	63.2 (6.9)	37.2 (13.3)	0.25	−1.19	1
Area under the curve (AUC) values							
Ghrelin, pg/mL × 180 min	121,448.8 (8043.0)	118,567.5 (10,924.9)	136,971.2 (12,734.0)	102,180.0 (11,838.1)	0.01	−2.53	0.26
PYY, pg/mL × 180 min	18,554.6 (1462.4)	17,075.3 (1559.3)	16,345.9 (963.8)	15,318.5 (2965.7)	0.88	0.15	1
GLP‐1, pmol/L × 180 min	3342.4 (1220.6)	1488.0 (271.8)	3228.8 (1556.0)	2409.6 (1075.4)	0.23	1.24	1
Glucose, mg/dL × 180 min	16,350.0 (310.9)	17,680.0 (1049.8)	16,095.9 (275.3)	16,712.5 (1592.0)	0.28	−1.11	1
Insulin, *μ*IU/mL × 180 min	11,197.1 (1349.9)	12,752.5 (1202.1)	5536.8 (568.4)	3945.0 (829.9)	0.12	−1.66	1
FFA, *μ*Eq/L × 180 min	85,135.6 (5505.3)	91,377.5 (9470.0)	107,840.3 (6442.8)	149,167.5 (8672.4)	0.02	2.37	0.43
TG, mg/dL × 180 min	23,580.0 (1866.9)	19,932.5 (2733.3)	16,885.6 (1364.0)	18,182.5 (2914.5)	0.06	1.20	1
Hunger, mm × 180 min	7999.7 (837.7)	9855.0 (2123.8)	9916 .0 (920.7)	6125.0 (2115.0)	<0.01	−3.20	0.13
Satiety, mm × 180 min	8761.9 (601.3)	6045.0 (2028.6)	7246.0 (580.2)	10,155.0 (2252.9)	<0.005	3.56	0.04
PFC mm × 180 min	8556.6 (824.0)	9684.0 (2258.0)	9412.0 (988.5)	4960.0 (2047.3)	<0.01	−2.88	0.14
*Ad libitum* lunch meal							
Energy, kcals	864.8 (67.3)	556.9 (78.7)	454.1 (63.4)	344.7 (103.4)	0.23	1.23	1
Food craving index (FCI) score							
Fasted	39.7 (2.7)	39.7 (5.9)	36.7 (2.2)	27.5 (5.1)	0.04	−2.15	1
Fed	37.4 (2.6)	41.3 (5.8)	36.47 (3.0)	30.3 (5.9)	0.04	−2.16	1

DIET, diet‐induced weight loss control group (*n* = 17); RYGB, Roux‐en‐Y Gastric Bypass (*n* = 6); AUC, Area under the curve; SE, Standard error (unadjusted); FFA, Free fatty acids; TG, triglycerides; PFC, Prospective food consumption.

**Figure 2:**
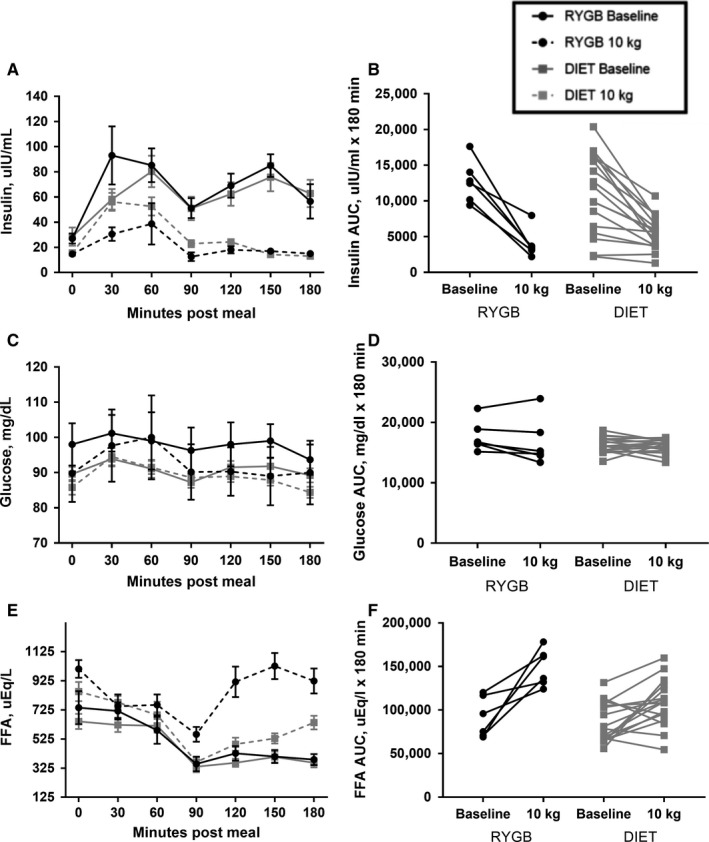
Metabolite response to breakfast test meal pre and post 10 kg of weight loss in participants undergoing RYGB or DIET. Curves for insulin (A), glucose (C), and FFA (E) are shown at 0 min and every 30 min for 180 min following the breakfast meal. Values are means ± SEM. Also shown are individual changes in AUC from pre to post 10 kg of weight loss for insulin (B), glucose (D), and FFA (F). RYGB, Roux‐en‐Y Gastric Bypass (*n* = 6); DIET, diet‐induced weight loss control group (*n* = 17); AUC, Area under the curve.

Group and individual glucose, insulin, and FFA response to the test meal are presented in Figure 2. No group by time interaction or group differences for fasting glucose, insulin, or FFA or AUC for glucose or insulin were detected (Table 2, all *P* > 0.05). However, the main effect of time was significant for a decline in fasting glucose and insulin, and insulin AUC (*P* < 0.01, *P* = 0.03, and *P* < 0.01, respectively), and an increase in fasting FFA (*P* < 0.01) in both groups from pre to post 10 kg of weight loss. The main effect of time for reduction in glucose AUC was marginally significant from pre to post 10 kg of weight loss (*P* = 0.07). For FFA AUC, a significant group by time interaction was detected (Table 2, *T* = 2.37, *P* = 0.02), with the increase from pre to post 10 kg of weight loss being greater in the RYGB group as compared to the DIET group. No significant group by time interaction or group differences were seen for fasting TG or leptin, or TG AUC (Table 2, all *P* > 0.05). Participants in both groups displayed a significant reduction in fasting leptin (*P* < 0.01) and a trend for a decrease in TG AUC (*P* = 0.06) from pre to post 10 kg of weight loss.

### Appetite ratings

**Figure 3:**
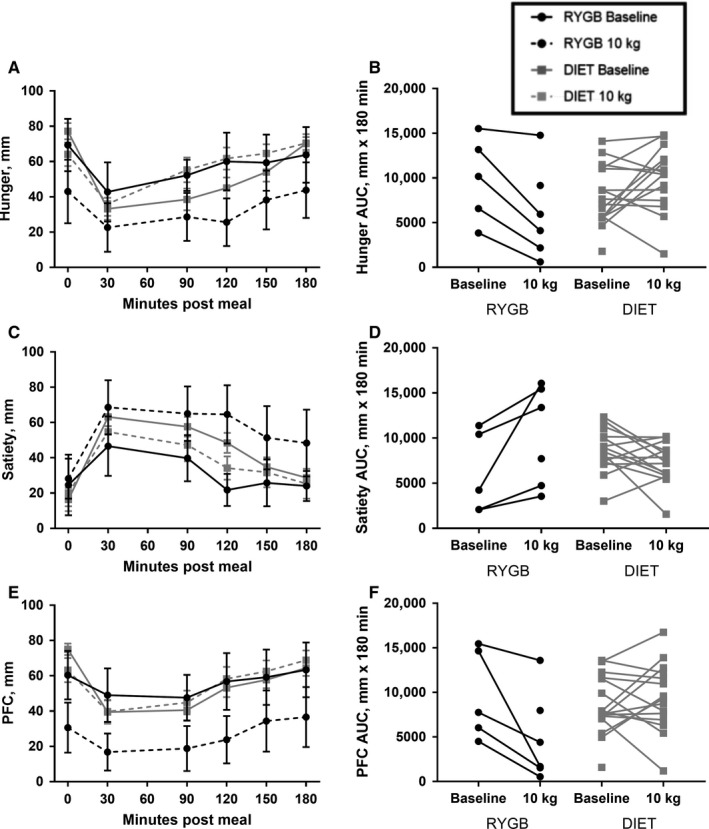
Subjective appetite response to breakfast test meal pre and post 10 kg of weight loss in participants undergoing RYGB or DIET. Curves for hunger (A), satiety (C), and PFC (E) are shown at 0 min and every 30 min for 180 min following the breakfast meal. Values are means ± SEM. Also shown are individual changes in AUC from pre to post 10 kg of weight loss for hunger (B), satiety (D), and PFC (F). Appetite ratings were evaluated with 100 mm VAS. RYGB, Roux‐en‐Y Gastric Bypass (*n* = 6); DIET: diet‐induced weight loss control group (*n* = 17); AUC, Area under the curve; PFC, prospective food consumption; VAS, visual analogue scale

Group and individual AUC for subjective appetite ratings (hunger, satiety, and PFC) are presented in Figure 3. Baseline appetite ratings were not collected on two participants (one in each group), and follow‐up appetite ratings were not collected on two participants (both in the DIET group). No group by time interaction or group differences for fasting hunger, satiety, or PFC were detected (Table 2, all *P* > 0.05). However, the main effect of time was significant for a decline in fasting hunger (*P* = 0.02) in both groups from pre to post 10 kg of weight loss. There was a significant interaction between group and time for hunger, satiety, and PFC AUC (Table 2, *T* = −3.20, 3.56, −2.88 respectively, all *P* < 0.01), with the RYGB group reporting decreases in hunger and PFC and increases in satiety, compared to the DIET group.

### Food cravings

Scores on the FCI in the fasted and fed states before and after 10 kg of weight loss are presented in Table 2. There was a significant interaction between group and time for food cravings in both the fasted and fed state (Table 2, *T* = −2.15 and −2.16, both *P* = 0.04), with the RYGB group reporting decreases in FCI scores, compared to no change in the DIET group.

### Visual stimuli evaluations

No group by time interactions or group differences were detected in VAS scale ratings of high– and low–hedonic food images in either the fasted or fed state (*P* > 0.05 for all); data not shown. In the fasted state, participants in both groups decreased their reported “appeal”, “pleasantness”, and “desire to consume” of both high– and low–hedonic visual stimuli from baseline to 10 kg of weight loss (all *P* < 0.05 for main effect of time). In the fed state, only “appeal” of high‐hedonic foods significantly decreased from baseline to 10 kg (*P* = 0.02 for main effect of time).

### *Ad libitum* energy intake

There were no significant group by time interactions for energy intake at the *Ad libitum* lunch meal (Table 2, *T* = 1.23, *P* = 0.23). Both the RYGB and DIET groups decreased energy intake from baseline to 10 kg (*P* < 0.01 for main effect of time), though baseline *ad libitum* energy intake was lower in the RYGB group (*P* = 0.04).

### Sensitivity analysis

The sensitivity analysis with males removed produced similar results with some slight attenuations in *P‐*values that are likely due to reduced sample size. One exception is VAS Hunger AUC where the treatment effect became insignificant (*P* = 0.06).

### Correlational analyses

Appetite measures were not significantly related to *ad libitum* energy intake (all *P* > 0.05). FCI scores in the fasted state was significantly associated with *ad libitum* energy intake (*P* = 0.01) such that a one unit increase in fasting FCI is associated with 9.75 more kcals consumed on average in both groups. Adjustment does not change the statistical significance of any of the findings regarding treatment effect; however, the difference in the treatment effect is increased approximately 25%. Given the sample size it is difficult to determine if this is a significant magnification of the treatment effect.

## Discussion

The present study was conducted to compare the effect of RYGB versus VLCD–induced weight loss on indices of appetite regulation and metabolic health. Results of this trial indicate that with matched weight loss, following the same VLCD, measures of appetite regulation are differentially altered between RYGB and DIET. Hunger, satiety, PFC, and food cravings change in a manner supportive of continued decreased energy intake in response to RYGB, but in a manner promoting increased energy intake in response to DIET. These findings, brought about by both anatomical and physiological alterations from surgery, might explain the greater success in long‐term weight loss maintenance in those who undergo RYGB as compared to those who lose weight with caloric restriction alone (Maciejewski et al. 2016).

Both RYGB and DIET groups experienced substantial reductions in fasting leptin values, as would be expected with significant weight loss (Benoit et al. 2004; Maclean et al. 2011). Interestingly, though, changes in subjective indices of appetite as well as appetite–related hormones differed between RYGB and DIET. Hunger, satiety, and PFC were altered in opposite directions following weight loss between the RYGB and DIET group, with decreased hunger and PFC and increased satiety reported following RYGB and increased hunger and reduced satiety reported by the DIET group in response to a test meal. These findings are in concordance with previous reports showing an anorectic appetite response following RYGB (le Roux et al. 2007; Bryant et al. 2013; Schmidt et al. 2016), with our findings extending this work by including a DIET control group with matched weight loss. These changes in appetite with RYGB have frequently been hypothesized to be due to altered gastrointestinal anatomy and nutrient delivery that change postprandial gut appetite‐related hormone responses (Moran 2009; Beckman et al. 2010). Our findings partially support this view. Participants in the DIET group experienced an increase in ghrelin AUC, while no change was seen in the RYGB group from pre‐ to post‐weight loss. Prior investigations have shown similar results of no change in ghrelin or a decrease in ghrelin in response to RYGB (Beckman et al. 2010; Harvey et al. 2010; Schmidt et al. 2016; Steinert et al. 2017), even when weight loss was not matched between surgical and diet‐induced weight loss modalities (Cummings et al. 2002). We did not see statistically significant changes to the appetite–related hormones PYY or GLP‐1 in either the RYGB or DIET group, which is in disagreement with other studies indicating that these appetite–related hormones may be increased with RYGB and decreased with DIET (Borg et al. 2006; Morinigo et al., 2006; Olivan et al. 2009; Beckman et al. 2010; Harvey et al. 2010; Schmidt et al. 2016; Steinert et al. 2017). This may be due to provision of a smaller absolute caloric load provided in the test meal following weight loss in the current trial as opposed to the same absolute, but greater relative caloric load provided in earlier investigations. However, we also compared only the post‐intervention meal responses between the DIET and RYGB groups and found no difference between them in the PYY and GLP‐1 responses to the same volume test meal (data not shown). In addition, variable individual responses are noted, which given the small sample size in the current trial, likely masked group changes seen in larger trials.

Despite changes to appetite indices that may promote reductions in energy intake with RYGB and increases in energy intake with DIET, *ad libitum* energy intake during the laboratory buffet meal as well as ratings of high‐ and low‐hedonic food images were similarly reduced in both groups. These findings differ slightly from findings by Nielsen et al. who showed no changes in food preference as determined by intake of various food categories as well as a picture display task before and 6‐months post RYGB, although in agreement with us and others, *ad libitum* intake of a buffet meal was decreased post‐op in their trial (Manning et al. 2015; Nielsen et al. 2017). This may be because our measurements were conducted while participants in both RYGB and DIET groups were still in a period of active weight loss and thus motivated to consume less food and view food images in a less appetizing manner due to experiencing success with weight loss. It is also possible that the decrease in *ad libitum* energy intake was also due to provision of a smaller caloric load and in liquid form post weight loss as opposed to the solid meal with larger calorie load presented at baseline. Similarly, the lack of sufficient power to detect group differences is also important to note.

However, food cravings, as assessed via the FCI questionnaire, were decreased in both the fasted and fed state for participants in the RYGB group, with no change noted in the DIET group. The changes in food cravings may be related to the altered gut appetite–related peptide response seen in the RYGB group, as gut peptides are not simply involved in homeostatic regulation of EI, but may also alter reward–based eating pathways and central appetite regulation (Malik et al. 2008; Harvey et al. 2010; Manning et al. 2015). For instance, in a cross‐sectional study utilizing functional magnetic resonance imaging (fMRI) technology, Sholtz et al. have shown less activation in brain–reward regions in response to viewing food images in participants who underwent RYGB as compared to those who underwent gastric banding or BMI–matched unoperated controls (Scholtz et al. 2014). Similarly, Ochner et al. reported decreased activation in brain regions involved in reward and inhibition before and 1 month after RYGB in female participants (Ochner et al. 2011). Future analysis should expand upon the current evidence by utilizing fMRI analyses to investigate pre‐ and post‐RYGB changes in comparison to relevant control groups (e.g., diet‐induced weight loss, pharmacotherapy induced weight loss, and never obese individuals).

As we are underpowered for the current trial, our results showing no group differences in fasting and AUC changes for glucose and insulin between groups cannot be interpreted to mean that weight loss, regardless of modality equivocally alters these metabolic health measures. Prior work comparing metabolic health outcomes between RYGB and DIET‐induced weight loss typically report superior outcomes with surgical interventions. However, these trials tend to be confounded by greater weight loss in the participants undergoing surgery compared to those attempting weight loss via lifestyle changes alone, and/or lack a weight–loss control group (Buchwald et al. 2004; Gloy et al. 2013; Khoo et al. 2014). Interestingly, a trial performed by Schmidt et al. comparing 11 weeks of diet‐induced weight loss versus 8 weeks of diet‐induced weight loss + RYGB and continued diet for 3 weeks, found no differences in various indices of glycemic control between the groups, despite greater weight loss in the RYGB group (Schmidt et al. 2016). This may be due to a large level of total weight loss (>13%) in both groups when measurements were conducted. While FFA AUC increased in both groups in response to weight loss, the increase was greater in the RYGB group compared to the DIET group, with the separation becoming most pronounced 120‐ min postprandially. A study by Johansson and colleagues comparing adults who had undergone RYGB to normal weight controls also showed increased FFA in the RYGB group at the same postprandial time as in our investigation (Johansson et al. 2007). These findings suggest that RYGB can lead to decreased inhibition of lipolysis earlier in the postprandial period. While speculative, this may be due to an increased rate of gastric emptying resulting from the smaller stomach created with surgery. The smaller stomach results in the food bolus taking up a larger percentage of the available space following surgery, thus increasing the speed of gastric emptying (Minami and McCallum 1984).

The current study has several strengths. First, this is one of the few trials that is a prospective comparison of appetite–related outcomes and metabolites between RYGB and VLCD. Second, we studied participants before and after matched levels of weight loss. This allowed for a true comparison of the method of weight loss on our outcome variables by avoiding the common limitation of greater weight loss in the bariatric surgery group seen in previous studies. Third, the inclusion of both hormonal and behavioral indices of appetite regulation extends prior work and provides greater information on mechanisms that may explain the enhanced weight loss maintenance typically seen with RYGB. Fourth, our design included a clinically and physiologically relevant test meal by adjusting the caloric load for weight loss and thus providing the same “relative” caloric load pre and post weight loss. Finally, diet was tightly controlled prior to assessment days.

Despite these strengths, several limitations must be discussed. First, the *ad libitum* lunch meal provided pre weight loss and post weight loss differed in form (solid food before weight loss vs. liquid after weight loss). However, like our decision to provide the same “relative” but different absolute caloric loads for the breakfast preload before and after weight loss, the difference in *ad libitum* meal type is also more physiologically and clinically relevant as it matches the dietary habits required of patients following the RYGB procedure. Nonetheless, differences in the lunch meals could have influenced total energy intake and contributed to the decreased *ad libitum* caloric intake seen from pre weight loss to post weight loss. Second, our sample size was small, particularly in the RYGB group and few men were included in the current trial. The goal was to enroll and obtain complete data on *n* = 16 participants per group. Due to unanticipated challenges we were unable to enroll this targeted number in the RYGB group. Therefore, we are underpowered for the primary outcomes, thus limiting our ability to discuss null results as truly representing no difference between groups. We are also unable to evaluate if men and women respond differently to RYGB or diet‐induced weight loss. Third, while weight loss was matched between groups, the time to achieve 10 kg of weight loss differed between groups. Therefore, it is possible that the shorter time until weight loss for the RYGB group compared to the DIET group influenced the appetite and appetite–related hormone responses (Coutinho et al. 2018). However, this difference in time to weight loss is likely unavoidable due to both the acute elevation in metabolic rate due to a surgical procedure (Long et al. 1979) and the anatomical alterations which make decreased caloric intake a requirement for the RYGB group. Fourth, participants were not randomly assigned to RYGB or DIET interventions. While unavoidable due to the nature of this study, this could have resulted in selection bias and inherent group differences. Fifth, we did not evaluate the menstrual status of women, nor did we complete measurements at the same phase of the menstrual cycle. This limitation is also unavoidable since we were scheduling patients for testing based upon surgical schedule (in the RYGB group) and time at which 10 kg weight loss was achieved. Sixth, we did not measure gastric emptying in our participants, which likely differed postintervention between RYGB and DIET groups and, as mentioned above, contributed to differences in postprandial appetite–related peptide and metabolite responses (Minami and McCallum 1984; Nguyen et al. 2014). We also did not measure ketone body production, which could have occurred in participants and played a role in the alterations seen in appetite indices (Nymo et al. 2017). However, the diets provided do not meet standard ketogenic diet guidelines (Freeman et al. 2007; Hall et al. 2016), nor has ketosis been observed in patients or research participants at our institution adhering to this same diet previously. In addition, this trial only studied one specific bariatric procedure (RYGB) and therefore our findings cannot be extended to other types of procedures. Furthermore, the total, and not active forms of appetite–related hormones were measured, which could explain some of the differences observed between our trial and prior investigations, and therefore future work will be necessary that measures the active form of these hormones. Finally, our trial only evaluated participants following acute weight loss, making us unable to determine if and how the changes that occurred, particularly in the appetite‐specific measures, relate to long‐term weight outcomes.

## Conclusions

In conclusion, the results of this study show that weight loss accomplished via RYGB resulted in changes to appetite–related hormonal and behavioral indices of appetite regulation in a manner that would, if maintained, likely promote decreased energy intake, and therefore sustained weight loss and improved metabolic profile. In comparison, matched diet‐induced weight loss resulted in changes in these indices that could promote increased energy intake and therefore weight regain and reversion of metabolic improvements seen with initial weight loss. These findings provide greater evidence into both homeostatic and hedonic appetite suppression mechanisms by which RYGB may result in enhanced weight loss maintenance versus traditional weight loss strategies. Healthcare professionals should counsel patients with obesity on changes to appetite–related indices that may occur with differing weight loss treatments.

## Conflict of Interest

None.
